# The Structure, Stability and Pheromone Binding of the Male Mouse Protein Sex Pheromone Darcin

**DOI:** 10.1371/journal.pone.0108415

**Published:** 2014-10-03

**Authors:** Marie M. Phelan, Lynn McLean, Stuart D. Armstrong, Jane L. Hurst, Robert J. Beynon, Lu-Yun Lian

**Affiliations:** 1 NMR Centre for Structural Biology, Institute of Integrative Biology, University of Liverpool, Liverpool, United Kingdom; 2 Protein Function Group, Institute of Integrative Biology, University of Liverpool, Liverpool, United Kingdom; 3 Mammalian Behaviour & Evolution Group, Institute of Integrative Biology, University of Liverpool, Leahurst Campus, Neston, United Kingdom; Instituto Butantan, Brazil

## Abstract

Mouse urine contains highly polymorphic major urinary proteins that have multiple functions in scent communication through their abilities to bind, transport and release hydrophobic volatile pheromones. The mouse genome encodes for about 20 of these proteins and are classified, based on amino acid sequence similarity and tissue expression patterns, as either central or peripheral major urinary proteins. Darcin is a male specific peripheral major urinary protein and is distinctive in its role in inherent female attraction. A comparison of the structure and biophysical properties of darcin with MUP11, which belongs to the central class, highlights similarity in the overall structure between the two proteins. The thermodynamic stability, however, differs between the two proteins, with darcin being much more stable. Furthermore, the affinity of a small pheromone mimetic is higher for darcin, although darcin is more discriminatory, being unable to bind bulkier ligands. These attributes are due to the hydrophobic ligand binding cavity of darcin being smaller, caused by the presence of larger amino acid side chains. Thus, the physical and chemical characteristics of the binding cavity, together with its extreme stability, are consistent with darcin being able to exert its function after release into the environment.

## Introduction

While urine provides a means for eliminating waste liquid from the body, many species also utilize urine as a vehicle to deposit species-specific scent signals in the environment. Whilst these scent signals are often low molecular weight biochemicals, mouse urine contains a polymorphic mixture of major urinary proteins (MUPs) [1] at very high concentration [2]. These are synthesised in the liver, secreted into serum and filtered efficiently into urine, with typical concentrations of 10–30 mg/ml in adult male house mice under laboratory conditions [3]–[5] and even higher levels under naturalistic conditions [6]. Both MUP production and scent marking are particularly elevated in male mice, with males excreting approximately twice as much MUP as females kept under similar conditions [5]–[7].

MUPs are detected directly by Vmn2r putative pheromone receptors (V2Rs) in the basal layer of the vomeronasal organ [8], [9]. In addition to this, MUPs bind low molecular weight hydrophobic organic compounds in a central calyx enclosed by an eight-stranded beta barrel [10]. Within mouse urine, these ligands include a number of known volatile pheromones such as the male-specific urinary volatiles 2-*sec*-butyl-4,5-dihydrothiazole, 3,4-dehydro-*exo*-brevicomin and 6-hydroxy-6-methyl-3-heptanone [11]–[13]. These act as reproductive priming pheromones, triggering hormonal responses and accelerating puberty in female mice [12]–[16]. When not bound to MUPs, these pheromones are extremely volatile and evaporate almost immediately from drying urine. The tight association between these volatiles and MUPs has the effect of modifying the release of volatiles from scent marks, extending the process over many hours. Thus, MUPs extend the time domain of volatile signals in urine scent marks [17]. MUPs, particularly those expressed in the nasal and vomeronasal mucosa, also are likely to be important for delivering hydrophobic urinary volatiles that are held in scent marks to receptors in the vomeronasal organ [18], [19].

MUPs are small 18–19 kDa lipocalins that are encoded by a large multigene cluster on mouse chromosome 4. In the most completely sequenced mouse genome (the C57BL/6J inbred strain, mouse genome database (MGI) [20]), there are at least 21 protein-coding genes, although several gaps in the genome within this region may harbor additional genes [21], [22]. These genes are divided into a central region that encodes a clade of about 13 proteins sharing very similar primary sequences (over 97% identical), flanked by peripheral regions that encode at least a further 8 proteins. The peripheral MUPs are substantially more diverse in primary sequence, sharing about 80% identity with each other and about 69% identity with central MUPs. The considerable similarity between MUPs encoded within the central region is due to multiple gene duplication events during recent rapid expansion of this region in the house mouse [21].

Most of the MUPs excreted in mouse urine are encoded by central genes, each mouse expressing a polymorphic and stable pattern of MUPs that differs from each other by a small number of amino acids [6]. Among wild mice, individual polymorphism is so great that unrelated mice express distinct individual patterns [23], [24]. This individual polymorphism provides an identity signal in urine scents that is used for individual recognition [23], [25]. By contrast, the sharing of MUP patterns between closely related individuals can also be used to recognize close kinship [26]. Signalling involves not only the proteins themselves but, because MUPs differ in their affinity for specific volatile ligands [27], the individual pattern of MUPs influences the pattern of urinary volatiles held [14].

In addition to the individual-specific pattern of central MUPs in mouse urine, we discovered another MUP (MGI MUP20), expressed only by male mice [28], that plays an important role as a male sexual attractant pheromone. Named darcin in recognition of its unique role [29], this MUP stimulates an instinctive attraction in females to spend time near male urine. Indeed, purified recombinant darcin is as attractive to female mice as intact male urine, while male urine without darcin fails to elicit female attraction. Even more significantly, contact with darcin stimulates strong and rapid associative learning such that females learn the same attraction towards the volatile airborne scent signature of the male [29]; without darcin, females fail to learn any attraction to a male's airborne signature and find this no more attractive than scent from another female. Mice also learn a preference for spatial cues associated with the location of darcin, a preference that is learned on a single encounter and remembered for at least two weeks [30]. Thus darcin plays a key role in attracting females to spend time in a male's scent marked area, stimulating females to learn where male scent marks are located and to become attracted to the individual odour of the male himself [30].

Darcin, which has a mature molecular weight of 18,893 Da (i.e. lacking the first 19 residue signal peptide) is effective as a pheromone on its own but also has unusual ligand binding properties. It is responsible for binding most of the male-specific pheromone, 2-sec-butyl 4,5 dihydrothiazole (SB2HT), one of the most abundant volatiles in male mouse urine, and provides a very extended release of this volatile ligand over many hours once urine is deposited in the environment [28]. Notably, darcin is not a central MUP but is encoded by one of the peripheral genes in the *Mup* cluster; these peripheral MUPs have much more diverse tissue expression and function than those encoded by central genes, which are all synthesized in the liver for urinary excretion [21], [31].

The unique function of the MUP darcin as a sex pheromone that stimulates both instinctive female attraction and learning provides an imperative to understand the structure and properties of this pheromone compared to other urinary MUPs. Here, we compare the three-dimensional structure and biochemical properties of darcin with a mature central urinary MUP, MUP11, and show that darcin has some unique properties that are highly relevant to its function.

## Methods and Materials

### Preparation and purification of recombinant darcin

Recombinant darcin and MUP11 were both expressed heterologously as N-terminal His_6_-tagged fusion proteins in *E. coli* using codon-optimised synthetic genes. The proteins were purified as described previously for darcin [32].

The MUPs were present in the soluble fraction of the bacterial cell lysate and purified by virtue of the hexahistidine tag by nickel affinity chromatography and dialysed against 50 mM sodium phosphate buffer containing 20 mM NaCl, pH7.4. This preparation was >95% pure (assessed by SDS-PAGE) and used without further purification. The intact masses of the proteins were determined by electrospray ionisation mass spectrometry and these were within 2 Da of the predicted mass (not shown).

### NMR spectroscopy

For NMR investigation, spectra were acquired at 300 K from a 1 mM darcin solution in 25 mM potassium phosphate, pH 6.8, containing 0.2% (w/v) NaN_3_ and 10% (v/v) [^2^H_2_]O on Bruker AVANCE II 600 MHz and 800 MHz spectrometers equipped with 5 mm triple resonance cryoprobes. Spectra were processed using Topspin2.1 (Bruker) and the Azara processing package provided as part of the CCPN suite with assignment carried out using CCPN Analysis [33]. Triple resonance assignment was obtained utilising two-dimensional HC and HN HSQCs in conjunction with standard three-dimensional triple resonance backbone and side-chain experiments [34]. Assignment of aromatic side-chain residues was made using 2D ^1^H-^13^C HSQC and homonuclear [^1^H] NOESY and TOCSY spectra recorded in both [^2^H_2_]O and H_2_O.

### Structure determination

The structural analysis of both MUPs were performed using CYANA 2.1 software [35], with input data of shift lists derived from ^1^H-^15^N- and ^1^H-^13^C HSQC spectra, along with un-assigned NOESY peak lists and additional restraints from 34 hydrogen bonds and 114 φ and ψ torsion angles produced by TALOS [36]. CYANA 2.1 was run with standard protocols using 7 cycles of automated NOE assignment and structural calculations, producing 100 structures per cycle. Structures were calculated using a total of 3146 (darcin) and 3833 (MUP11) unambiguous interproton distance restraints. A final ensemble of the best 20 water-refined structures was selected on the basis of low RMSDs, low NOE energies, and was validated with PROCHECK-NMR [37] using the iCing interface (http://nmr.cmbi.ru.nl/icing/iCing.html) [38].

### Structure analysis

Secondary structure of darcin and MUP11 NMR structures were calculated using STRIDE webserver [39], [40]. Surface analysis used NACCESS (Hubbard,S.J. & Thornton, J.M. (1993), ‘NACCESS’, Computer Program, Department of Biochemistry and Molecular Biology, University College London) for identification of exposed hydrophobic residues, CCP4MG [41] for calculation and displaying electrostatic surface potentials and Pymol (The PyMOL Molecular Graphics System, Version 1.3, Schrödinger, LLC) for secondary structure and side chain analysis. In addition comparative analysis of the MUP family employed PROMALS3D [42], [43] for secondary structure driven sequence alignment and Multiprot [44] for homologous structure alignment. Random coil index (RCI) analysis was through the RCI webserver [45]. The CASTp programme [46] was used to measure cavity size and the programmes PIC [47], PDBePISA [48] and LIGPLOT [49] measured intramolecular hydrophobic and ionic contacts.

### Relaxation analysis

Relaxation data was collected at 600 MHz. T_1_ delays of 4, 10, 20, 40, 60, 80, 120, 160, 220, 280, 340, 60 ms and T_2_ delays of 1, 2, 4, 8, 12, 16, 20, 30, 40, 60, 80 ms were recorded using standard Bruker pulse sequences. Heteronuclear ^15^N{^1^H} NOEs were also collected. Relaxation curves were determined using CCPN analysis software [33].

### MUP Titrations (Urea, menadione, Vitamin K3 (K3), N-phenyl naphthylamine (NPN), 2-sec-butyl-thiazole (SBT))

^1^H-^15^N HSQC NMR titration experiments were performed on a Bruker Avance 800 MHz spectrometer equipped with a 5 mm cryoprobe at an experimental temperature of 298 K. Urea was titrated from zero to a final concentration of 7.5 M in 0.2 mM protein and allowed to equilibrate for no less than 30 minutes prior to acquisition of spectra. The peaks in each ^1^H-^15^N HSQC were assigned in the CCPN software package. Analysis and the percentage disappearance were calculated for each titration point. Peaks that overlapped or were unresolved due to crowding were excluded from the analysis.

Ligand titrations were carried with NPN (Sigma, UK) and K3 (Sigma, UK) dissolved in MeOH; commercially available SBT was used as neat liquid (Endeavour Specialty Chemicals, UK). It should be noted that this ligand is *not* the native *2-sec-butyl-4,5dihydrothiazole but the oxidised analogue*. To distinguish clearly between these similar molecules, we refer here to the native ligand in male mouse urine as SB2HT; the reader is cautioned that SBT has been used in some previous publications as an acronym for the native ligand [50]. Reference spectra of MUP11 and darcin collected in 5% (v/v) MeOH showed no major changes in chemical shifts, indicating that this level of organic solvent had minimal effects on protein structure. Ligands were titrated in small volumes (1–5 µL) into the protein solution at high concentration (50 mM) to ensure final concentration of MeOH did not exceed 5%. The peaks in each HSQC were assigned in the CCPN software Analysis. Maximum change in chemical shift is calculated based on combination of proton and nitrogen chemical shift change: Δδ = {(ΔH)^2^ + (0.15ΔN)^2^}^1/2^.

### Isothermal titration calorimetry measurements

SBT binding was investigated using isothermal titration calorimetry (ITC). The experiments were carried out on purified samples of protein exchanged into 25 mM phosphate buffer containing 25 mM NaCl using a NAP25 desalting column (GE Healthcare). The SBT (supplied as neat liquid) was diluted in identical buffer. Control experiments whereby the SBT was titrated into buffer and buffer into protein exhibited no detectable heat exchange, confirming that there was appropriate match of buffer conditions with no evidence of dilution effects. To maintain consistency between titrations the same stock buffer was used in all protein and SBT preparations. Experiments were conducted at 25°C with an ITC_200_ (GE Healthcare) with a 40 µl syringe volume and 200 µl cell capacity. Titrations were carried out using between 40 µM and 100 µM of protein in the cell and a tenfold concentration of SBT (between 400 µM and 1 mM). SBT was added into the cell in sequential 1 µl injections (at a rate of 0.5 µl per second) with a 180 second interval between each injection. One site (three parameters) curve fitting was carried out using the MicroCal-supported ITC module within Origin version 7.

### Databank accession codes

Chemical shifts assignment of darcin and MUP11 are deposited in the BioMagResBank; Accession numbers 16840 and 17447 respectively. Atomic coordinates and NMR restraints of darcin and MUP11 have been deposited in the Protein Databank under the accession codes 2L9C and 2LB6 respectively.

## Results

**Figure 1:**
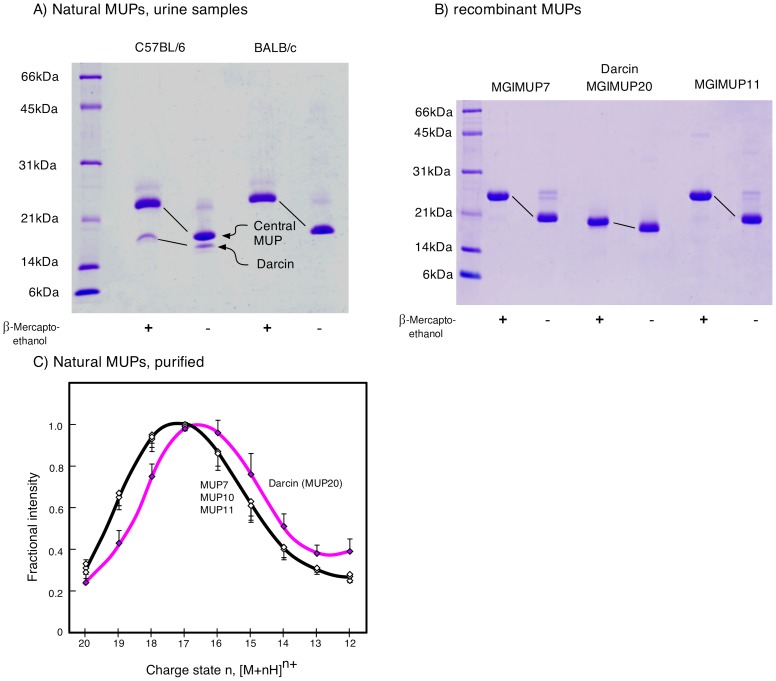
Anomalous properties of darcin compared to central MUPs. The peripheral MUP darcin (molecular weight 18893 Da) exhibits high mobility on SDS-PAGE compared to other urinary MUPs (molecular weight 18645–18708 Da). (A) Under non-reducing conditions, darcin (labelled) is readily resolvable from other urinary MUPs (major band). On reducing SDS-PAGE, darcin and other MUPs migrate more slowly, although the effect is much less pronounced for darcin. This is consistent with darcin retaining a more compact structure under reducing gel conditions. Urine samples were analysed from males of two mouse strains, C57BL/6, which express darcin, and BALB/c, which do not express darcin. (B) The same behaviour is evident for three recombinant MUPs, darcin, central MUP7 and central MUP11, with Darcin travelling further on the gel than other MUPs. (C) Darcin also exhibits anomalous behaviour under conditions of electrospray ionisation mass spectrometry. Compared to three central MUPs, darcin exhibits a different distribution of multiply protonated ions, tending to a lower charge state, despite having the same number of protonatable sites. This distribution of charge states may reflect the lower accessibility of some basic sites because darcin retains a degree of structure in the gas phase.

Darcin exhibits physicochemical properties that set it apart from other MUPs. Both the native protein from mouse urine and the *E. coli* expressed recombinant protein migrate at a higher mobility on SDS-PAGE, travelling further on the gel, than other MUPs (Fig. 1A, B). This enhanced mobility of darcin (in reduced or oxidised forms) is consistent with it retaining a more compact, partially collapsed structure that can penetrate the gel matrix more easily. When darcin and other central MUPs (with the same number of protonatable sites) are subjected to electrospray ionization mass spectrometry, the charge state distribution of darcin is skewed towards a lower degree of protonation than other MUPs (Fig. 1C), consistent with a compact structure that is not completely unfolded during the conditions of electrospray ionisation that were used.

To explore the differences between darcin and central MUPs, we solved the NMR structures of darcin (MGI MUP20) and a central MUP, MGI MUP11, under identical solution conditions. MUP11 encodes the same mature protein sequence as four other MUP genes (MGI nomenclature MUP9, 15, 18, 19). This protein, of average mass 18,694 Da, is commonly present in urine of inbred [11], [51] and wild mice [21], [29] and is expressed by both sexes unlike the male-specific darcin.

### Overall structure of darcin and MUP11

The assignment of darcin and MUP11 were, respectively, 97.3% and 91.9% of all expected ^1^H- ^13^C and ^1^H-^15^N chemical shifts (excluding the N-terminal purification tags and exchangeable side chain protons).

**Figure 2:**
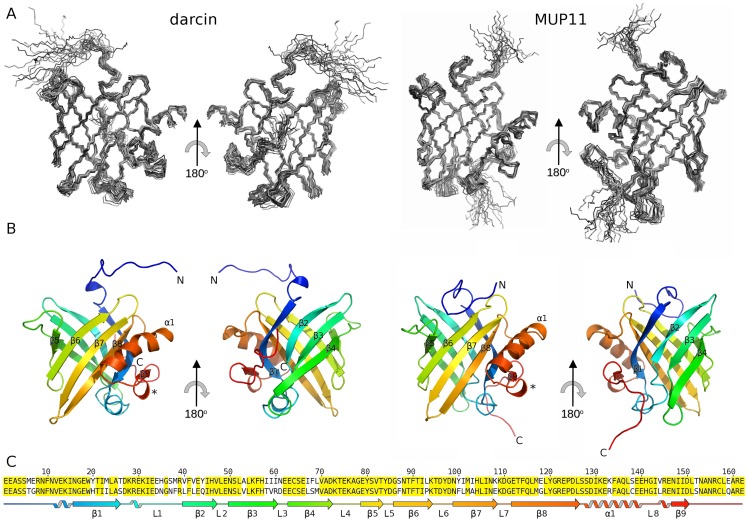
Solution structure of darcin (left) and MUP11 (right). For clarity 180° representations are shown. (A) The ensembles each comprise 20 lowest-energy models. (B) For each ensemble a representative closest-to-mean structure was selected and shown as a cartoon representation of the structural elements. Marked in asterisk is the conserved 3_10_-helix between α1 and β9. (C) Alignment of the primary sequence of darcin (top) with MUP11 (bottom) with conserved residues highlighted in yellow. The structural schematic for darcin is coloured to correlate with the colouring on the cartoon representation shown in (B), from N to C terminus as blue to red. In (C), the S-S bridge between C64 and C157 is indicated as black lines linking the two residues.

Notably, residues from the N-terminal region of darcin gave poor spectral quality: residues E7 and R8 resonances were unobservable in the ^1^H-^15^N HSQC, possibly due to intermediate chemical exchange as a result of structural heterogeneity. Severe resonance overlaps led to poorly-defined long-range NOEs for this region. For MUP11, however, it was possible to resolve resonances of all the N-terminal residues, with long-range NOEs observable in many cases. In contrast, definition of the C-terminal region was possible in darcin but not MUP11. For darcin the C-terminal region is defined by 20 long range distance restraints (i.e. restraints between residues separated by more than five amino-acids) involving residues L158, A160 or R161. On the other hand, the MUP11 C-terminal region is restrained by one long range NOE involving L158. All other NOEs from C-terminal residues (L158-E162) were either intra-residual or between adjacent amino acids, resulting in the C-terminal of MUP11 being structurally less well defined than darcin (Fig. 2A). The structural data of the N-terminal region obtained here are in general agreement with other MUP forms where the N terminal region is predominantly random coil in the NMR structures and absent in x-ray structures inferring a high degree of flexibility.

The ensemble of twenty lowest energy models of darcin and MUP11 each have, respectively, backbone RMSD values of 0.35 Å and 0.34 Å (Table 1). The NMR structure ensembles of darcin and MUP11 are very similar, showing a RMSD of 1.4 Å between the two ensembles for backbone residues 8-155. Both structures adopt the classical lipocalin fold, each forming an eight antiparallel-stranded beta barrel (labelled β1-β8 in Fig. 2B) with highly polar surface enclosing a hydrophobic core or calyx. β5 is considerably shorter than the other strands that make up the beta barrel; in MUP11, β5 is in fact poorly formed (Fig. 2B,C). The beta barrel is distorted by β9 being separated from the core barrel by the presence of a four-turn alpha helix α1, which runs parallel to β7 and β8. In addition to this large alpha helix (α1), there is one conserved 3_10_ helix between α1 and β9. Details of the secondary structure elements for the two MUPs are summarised in Table 2. Darcin (but not MUP11) also has two further 3_10_ helices, one near the N-terminus and the other located within loop L_1_. There is also an additional 3_10_ helix observable near the C-terminus in 35% of the darcin ensemble.

**Table 1:** Structural statistics for the refined NMR structures of darcin and MUP11.

*NMR constraints*		darcin	MUP11
	Total number of distance constraints	3148	3833
	Short range (|ij|≤1)	1592	1883
	Medium range (1<|ij|<5)	359	595
	Long range (|ij|≥5)	1197	1355
***Structure statistics (20 structures)***			
	Average number of NOE violations> 0.3Å	0	0
	NOE violations> 0.3Å	0	0
	Maximum NOE violation	0	0
***Ramachandran statistics***			
	Residues in most favoured regions	81.9	81.1
	Residues in additional allowed regions	17.9	17.3
	Residues in generously allowed regions	0.0	1.0
	Residues in disallowed regions	0.2	0.6
***RMS Deviations*** * ***from the mean structure***			
	Protein backbone (residues 12-152)	0.35Å	0.34Å
	Protein heavy atoms (residues 12-152)	1.00Å	0.98Å

*Quoted Root-Mean-Square Deviation (RMSD) is derived from comparison of closest-to-mean structure; i.e. representative structure, of each ensemble.

**Table 2:** Structural features of Darcin and MUP11(a).

Structural feature	MUP11	Darcin	Strand annotation	Inter-strand loop(b) and number of residues in loop	
3_10_-helix		V12 - K14			
β-strand	T21 - S26	Y20 - T26	β1	L_1_, 13aa	
3_10_-helix		R34 - K36			
β-strand	F41 - V47	F41 - V47	β2	L_2_, 3aa	
β-strand	L52 - H57	S51 - H57	β3	L_3_, 9aa	
β-strand	E66 - D72	I67 - K73	β4	L_4_, 5aa	
β-strand	E79 - V82	E79 - T83	β5	L_5_, 3aa	
β-strand	N88 - T95	S87 - T95	β6	L_6_, 4aa	
β-strand	F100 - K109	Y100 - K109	β7	L_7_, 2aa	
β-strand	E112 - G121	E112 - G121	β8	L_8_, 6aa	
α-helix	S128 - C138	S128 - H141	α1	L_9_, 4aa	
3_10_-helix	E139 - H141				
3_10_-helix	R145 - N147	R145 - N147	*(c)		
β-strand	Ile148 - Asp150	Ile148 - Asp150	β9		

(a) As defined by the programme Stride [40].

(b) Loop nomenclature: L_1_ (between β1 and β2), L_2_ (β2-β3), L_3_ (β3-β4), L_4_ (β4-β5), L_5_ (β5-β6), L_6_ (β6-β7), L_7_ (β7-β8), L_8_ (β8-β9), L_9_ (α1-β9).

(c) Conserved C-terminal 3_10_-helix.

**Figure 3:**
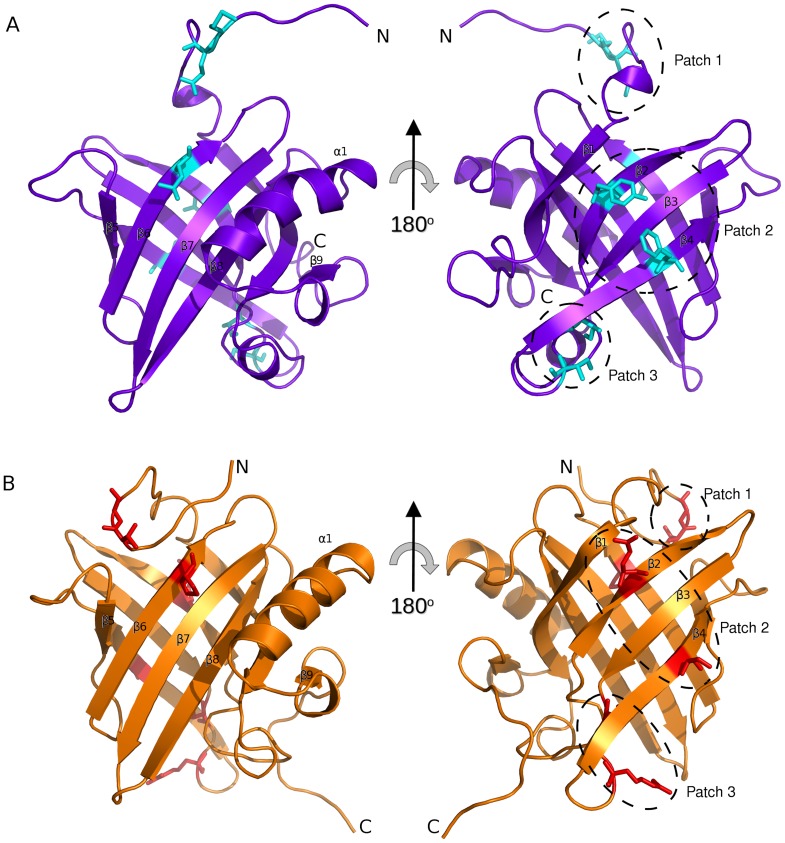
Schematic of MUP beta-barrel and inter-strand loop arrangement. Top left: top-down view of the beta barrel. Loops at the top, N-terminal end of the barre**l** are highlighted in green and magenta. Top right: bottom-up (C-terminal end,) view of the beta barrel. Loops at the bottom, C terminal end of the barrel are highlighted in blue and tan. Bottom: Alignment of darcin and MUP11 sequences with paired loop residues used to measure inter-loop distances highlighted, each residue pairs are coloured green, magenta, tan and blue in accordance with the schematic views.

Short hairpin loops L_2_, L_4_, L_6_ and L_8,_ with three to six amino acids in lengths, are located at one end of the β-barrel (the N-terminal, top end, Figs. 2B and 3), whereas the larger loops, L_1_, L_3_, L_5_ and L_7_ are found at the other end (C-terminal, bottom end, Figs. 2B and 3). Table 3 shows the closest distances between opposing loops at both ends of the β-barrel for darcin and MUP11. The closest distance is between L_1_ and L_5_, as in other MUP structures [19], [52] In addition, L_1_ appears to occlude/restrict access to the bottom, C terminal end of the internal hydrophobic cavity. The C-terminal region of these proteins comprises of the 3.5 turn α-helix that runs alongside the outward face of the β-barrel and is stabilised by hydrophobic interactions between I130, F134 and L137 of the helix and Y/F100, M102 of β7 and F114, M117 of β8. Beyond the final beta strand are 12 amino acids of the C terminal region that are anchored to L_3_ via a conserved disulphide bond between C157 and C64 (in β4).The incorporation of the disulphide bond was determined by ^13^C-β Cystine chemical shift characteristic of oxidised cystine (and structure calculations omitting cystine restraints resulted in structures that brought the two cysteines within range of disulphide bond formation). An additional cysteine present only in MUP11 (C138), was shown by DTT and SDS PAGE analysis not to play a role in dimer formation, as it is clearly buried on the internal face of α-helix 1.

**Table 3:** Closest opposing inter-loop distances (in Å) in Darcin and MUP11(a).

Loop Pairs	Darcin	MUP11
**N-terminal top end**		
L_4_ (G78 Cα) – L_8_ (R122 Cα)	21.95	22.32
L_2_ (L48 Cα) - L_6_ (Y97 Cα)	18.46	17.15
**C-terminal bottom end**		
L_1_ (V/L38 Cα) – L_5_ (D85 Cα)	10.18	10.99
L_3_ (I/T58 Cα) - L_7_ (D110 Cα).	21.04	21.00

(a) Distances measured between residues/atoms in brackets, using the most representative structure (Structure 1 in ensemble). First residues from darcin; second from MUP11.

**Figure 4:**
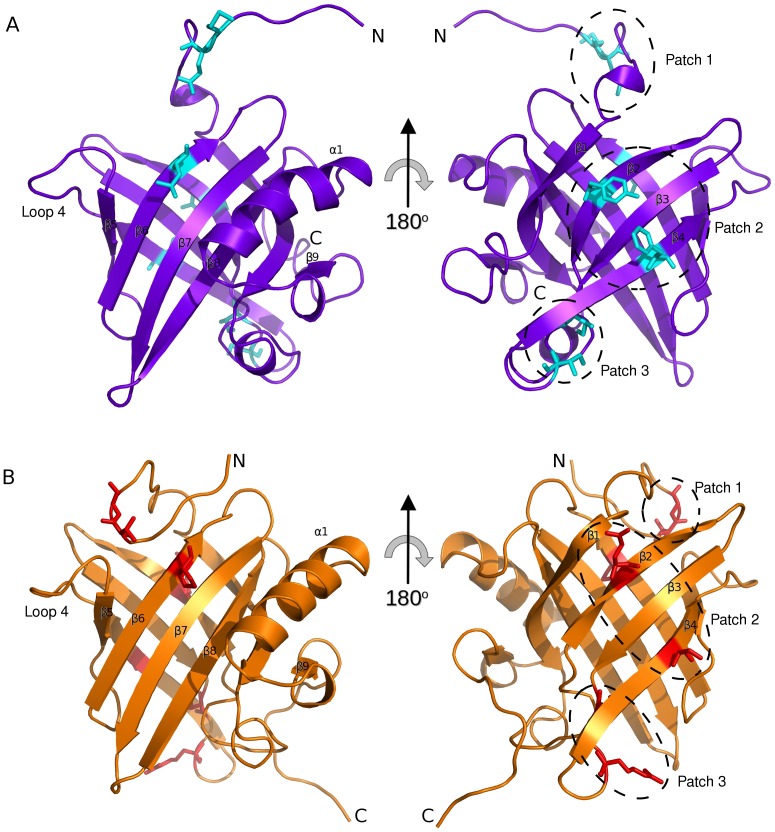
Variation in surface amino acids between darcin and MUP11. Darcin (mauve) (A) and MUP11 (orange) (B) are shown in the same orientations. Non-conserved surface exposed residue side-chains are shown as stick representations and shaded cyan (darcin) and red (MUP11). Only variations of residues that do not confer similar properties (polar, hydrophobic, charged, aromatic etc.) are shown, as Patches 1, 2 and 3 (see text). For clarity hydrogen atoms are omitted from the stick-representations of the residues shown.

The solvent exposed surface of both proteins is predominantly polar with similar surface charge distribution. Variation in the amino acid composition is limited to subtle changes in residue type that conserve the surface properties (i.e. V for L). However, three patches on the surface can be identified with divergent properties (Fig. 4). Patch 1 is the N terminal unstructured region which, in MUP11, forms contacts with loop 4 as defined by four NOEs involving S4 with P93, and twenty-one NOEs between T95 and E2, A3, S4, S5, N9, and F10 (Fig. 4); there are no equivalent NOEs observed in darcin. Patch 2 occurs on the side of the barrel comprising residues Y44 (β2), F68 (β4) in darcin (Fig. 4A) and Q44 (β2), S68 (β4) in MUP11 (Fig. 4B). Patch 3 is formed by I58 to I60 in darcin and by T58 to R60 in MUP11. The absence of patch 1 in darcin is possibly due to the larger bulkier nature of residues M6, E7 and L93, compared with the corresponding residues of T6, G7 and P93 in MUP11. In darcin, the patches 2 and 3 are predominantly hydrophobic, whereas in MUP11 these patches are polar. The differences of the solvent exposed surfaces in the different MUPs are not widely reported although the N terminal region is postulated to form a ‘lid’ in many lipocalin-type structures [10]; the variance of amino-acid type could confer function and/or specificity.

### Relaxation and dynamics

The beta barrel structure is well-defined, with a high degree of rigidity in both cases exhibiting only a small degree of flexibility in the majority of the loop regions as seen in NMR relaxation measurements and also random coil index (RCI) analysis (Fig. S1 in File S1). The rigidity of these hairpin beta loops is not surprising given that they comprise 2 to 6 amino acids each. Minor differences can be identified at the fourth loop (L_4_); in MUP11, this loop region exhibits NOE contacts to T6 and G7 of the N terminal region whereas in darcin, no equivalent interaction can be observed between the same loop and M6, E7 of darcin making L_4_ in darcin appear more mobile. The heteronuclear NOEs confirms the ensemble analyses data in that the C terminal region of MUP11 is more flexible than darcin (Fig. S1 in File S1).

### Hydrophobic pocket

**Figure 5:**
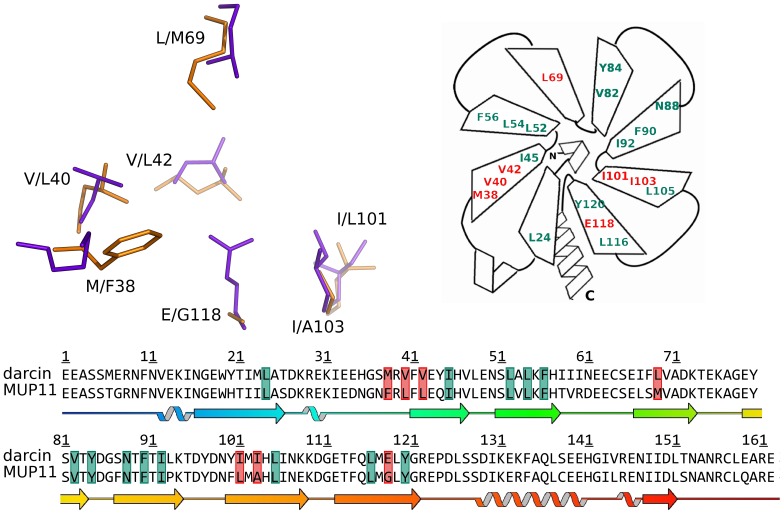
Comparison of binding cavities of darcin and MUP11. Left: Overlay of binding residues of darcin (mauve) and MUP11 (orange), where the differing amino acid residues in both darcin/MUP11 are labelled. Right: schematic of residues highlighted as part of the SBT binding site, conserved residues between darcin and MUP11 are green and variable residues are coloured red with the darcin residue only indicated. Bottom: Aligned sequences with SBT binding residues highlighted using the same colour scheme as above (conserved  =  green; variable  =  red), secondary structure schematic is aligned below the sequences with identical colour scheme to Fig. 2.

The core of the barrel is lined with predominantly hydrophobic amino acids that may be considered to be positioned in the centre of the barrel or pointing into the core of the barrel from either the N terminal, top or the C terminal, bottom end of the barrel. There are seven amino acids at the centre of the barrel: L42 (MUP11)/V42 (darcin) (β2); L54 (β3); M69 (MUP11)/L69 (darcin) (β4); V82 (β5); F90 (β6); A103 (MUP11)/I103 (β7) and G118 (MUP11)/E118 (darcin) (β8) (Fig. 5). There are eight residues lining the base of the barrel: L26 (β1); F38 (MUP11)/M38 (darcin) (β2); L40 (MUP11)/V40 (darcin) (β2); F56 (β3); Y84 (β5); N88 (β6); L105 (β7); L116 (β8). There are five residues lining the top end of the barrel: I45 (β2), L52 (β3), I92 (β6), L101 (MUP11)/I101 (darcin) (β7) and Y120 (β8) (Table S1 in File S1 and Fig. 5). Space filling models show that the hydrophobic core is protected from the solvent by conserved polar residues D85, K109 and D110, together with N61, E62 and S37 for darcin and R60, D61 and N36 for MUP11.

### Isothermal titration calorimetry

**Figure 6:**
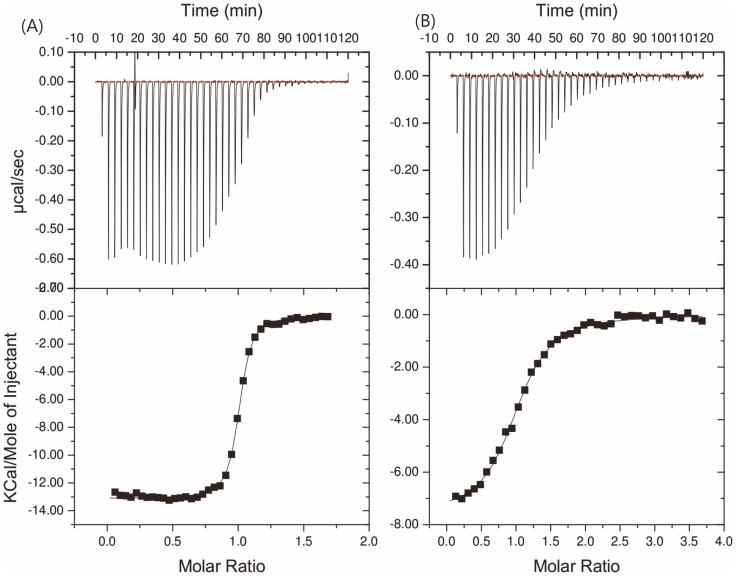
Isothermal titration calorimetry curves. Plots showing 2-*sec*-butyl thiazole (SBT) binding to darcin and MUP11 in 25 mM PO_4_^3−^, 25 mM NaCl, 298 K curve fit to a one-site model. (A) Darcin binds SBT with N (stoichiometry ratio)  =  1.0, K_D_∼0.173 µM, ΔH = **∼** -13.1 kcal/mol and TΔS = ∼3.9 kcal/mol. (B) MUP11 binds SBT with N (stoichiometry ratio)  = 1.0, K_D_ ∼2.76 µM, ΔH ∼-9.8 kcal/mol and TΔS = ∼ 2.2 kcal/mol.

Darcin and MUP11 bind SBT with, respectively, dissociation constants K_D_∼0.173 µM and ∼2.76 µM (Fig. 6). The overall thermodynamics for binding to darcin and MUP11 are dominated by favourable enthalpy, with small unfavourable entropy in both cases. The enthalpy of binding to MUP11 (ΔH∼-9.8 kcal/mol) is significantly less favourable than binding to darcin (ΔH∼-13.1 kcal/mol). The entropy TΔS for binding MUP11 is only marginally more favourable than that for darcin binding (-TΔS (MUP11) ∼2.2 kcal/mol vs -TΔS (darcin) ∼3.9 kcal/mol). The favourable enthalpy results from the binding of the SBT in the cavity rather than to a greater loss of degrees of freedom in protein upon binding; this is supported by evidence from structural studies for other MUPs where there appears to be only minimal changes in protein conformation upon pheromone binding [52]–[55]. These unusual thermodynamics that are characteristic of the hydrophobic interactions between MUPs and pheromones are well-documented and classified as “non-classical” hydrophobic interactions, unlike the classical ones which have favourable entropic contributions to the binding [52], [55]–[59].

SBT binds to MUPs in the occluded hydrophobic cavity. Crystal structures of MUP10 (PDB 1I06; annotated as MUP-I in [60]) and MUP4 (PDB 3KFF; annotated as MUP-IV in [19]) with SBT show that the pheromone binds via hydrogen bonds. In the case of MUP4, a direct hydrogen bond is formed between the SBT ring nitrogen and the carboxyl side-chain of E118 whereas in the MUP10, with a glycine residue in position 118, the hydrogen bond between the protein and the same nitrogen is through water molecules. This direct versus water-mediated hydrogen bond is one factor contributing to a ΔΔG of 1.9 kcal/mol in favour of MUP4 binding to SBT [19]. We observe a similar scenario here where substitution of E118 in darcin for G118 in MUP11decreases the affinity by 20-fold, with ΔΔG ∼-1.6 kcal/mol in favour of darcin; a glutamate residue in position 118, therefore, favours binding due to its ability to form a direct hydrogen bond with the ligand.

### Ligand binding cavity analysis

**Figure 7:**
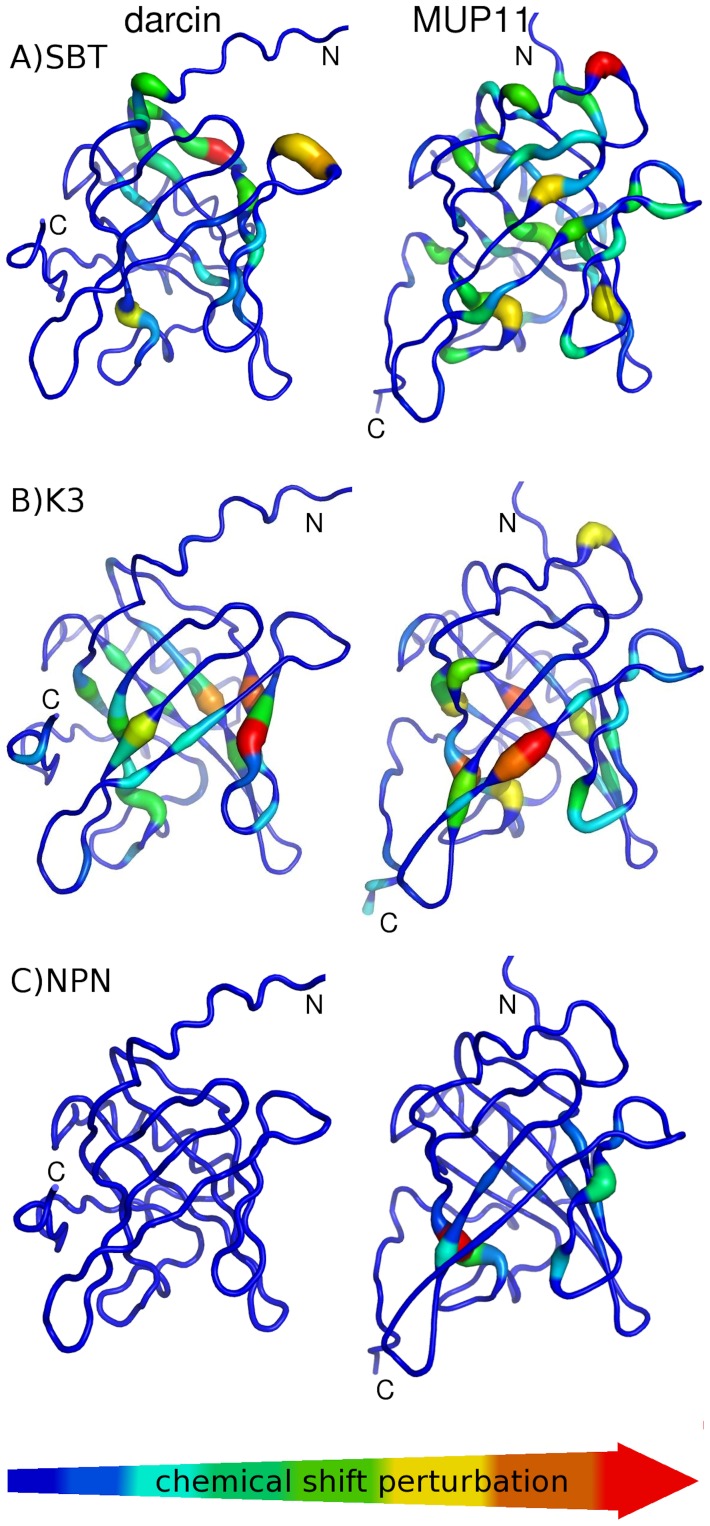
MUP cavity analysis. A binding cavity consensus was determined based on the active ligands identified by LigPLOT [49] and PDBePISA [48] in over 50% of the complex structures and shown mapped (in yellow) onto darcin (2L9C) and MUP11 (2LB6) (top two sequences). All ligand:MUP complexes available in the PDB are analysed. Residues identified as part of the binding site are highlighted according to ligand type: aromatic/pyrazole ligand (blue), aliphatic/non-cyclic molecule (orange).

Throughout the MUP protein family, there is overall conservation of the cavity-forming residues; these are identified using the PDBePISA analysis of existing MUP:ligand complex structures available in the Protein Databank. Between 14 and 18 residues predominantly define the binding cavity (Figs. 5 and 7). Analyses of the structures of both darcin and MUP11, using programmes such as CASTp confirm that these residues indeed form the binding cavity of both proteins.

**Figure 8:**
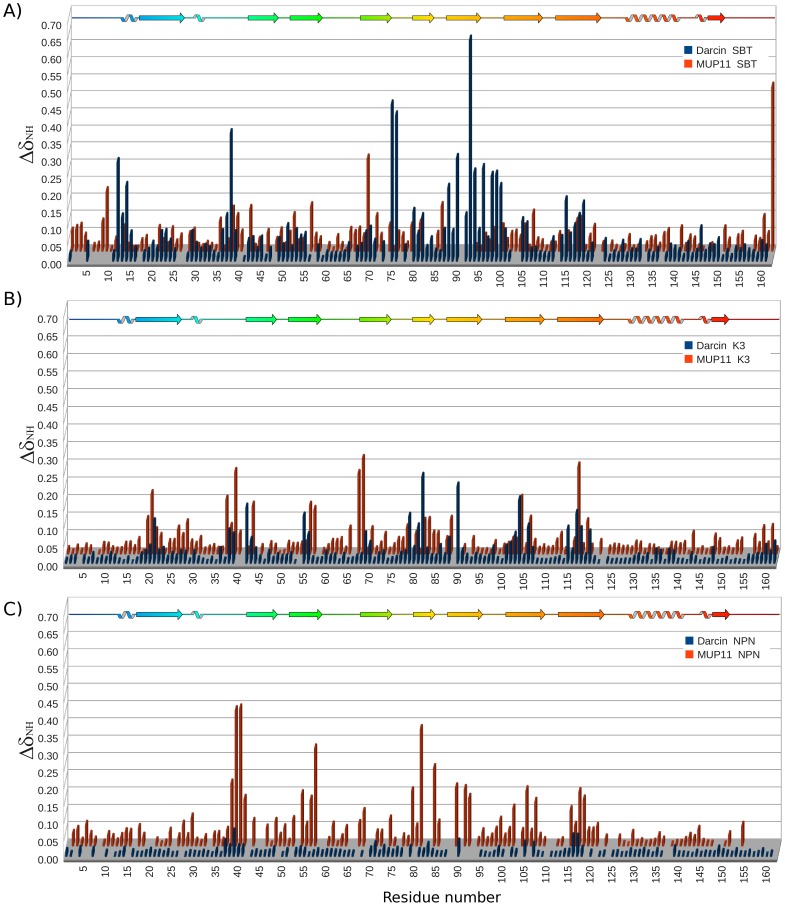
Ligand binding analysis by NMR. Histogram of chemical shift perturbations induced in darcin (blue) and MUP11 (orange) in the presence of at least five molar excess of SBT (A) K3 (B) and NPN (C). The secondary structure elements are represented by the schematic of darcin along the top of each plot, colour-coded as shown in Figure 2. All three ligands induced similar profiles of chemical shift changes with the most significant shift changes being observed for residues that form part of the pheromone-binding hydrophobic cavity. The exception being darcin with NPN which did not exhibit any combined backbone NH chemical shift changes above the cut-off threshold of Δδ = 0.15. Differences in the shift changes between the different complexes may be attributed to differences in affinities and/or residue composition of the binding cavity. The chemical shift perturbations (CSP) for non-overlapped resonances are calculated using the equation Δδ = {(ΔH)^2^ + (0.15ΔN)^2^}^1/2^ where ΔH and ΔN are, respectively, shift changes in the ^1^H and ^15^N dimensions.

The NMR chemical shifts of many residues of darcin and MUP11 are affected by the presence of SBT, many of which are from the conserved residues that form the binding cavity (Fig. 8). Additionally, in these studies, we use the chemical shift perturbations of the ^1^H-^15^N HSQC spectra for assessing whether a ligand binds. SBT, K3 and NPN, are known ligands of MUPs and occupy a volume of, respectively, 132.5, 158.1 and 175.8 Å^3^. The smaller ligands, K3 and SBT, bind to both darcin and MUP11 in the hydrophobic cavity of the beta barrel (Fig. 8). The larger NPN bound MUP11 (and other MUPs [27]) but not darcin. For darcin itself there are differences in the details of the residues affected by SBT and K3 which could be explained by both the size and increased conformational flexibility of the larger K3 ligand.

To further analyse the ligand cavity, the CASTp programme was used to obtain cavity areas and volumes, and to identify residues that form contacts with a spherical probe were performed. Using the default probe size of 1.4 Å and the best representative structure of the NMR structural ensemble, the cavity volume for darcin is 435 Å^3^, and for MUP11 490 Å^3^ making the darcin ligand cavity 11% smaller than the MUP11 cavity. The surface areas and total volumes for the two proteins are comparable (10667 Å^2^ and 21516 Å^3^ for darcin; 10007 Å^2^ and 21071 Å^3^ for MUP11). The number of residues that contact the probe is slightly larger for MUP11 than darcin (22 vs 19). Interestingly, as the probe size increases from 1.4 to 1.7 Å, the number of residues contacted by the probe decreases sharply for darcin whereas for MUP11, the decrease is less pronounced. To verify the method, this analysis was performed for other MUPs (limited to four unique protein sequences); both the peripheral MUPs, darcin and MUP4, stand out with a significant decrease in the number of contacts with the size of the probe, while structures of the only other central MUP in the Protein Databank, MUP10, show similar characteristics to MUP11. This computation analysis agrees with the experimental NMR data described above in which there is an upper limit for the size of pheromone that can bind to darcin.

**Figure 9:**
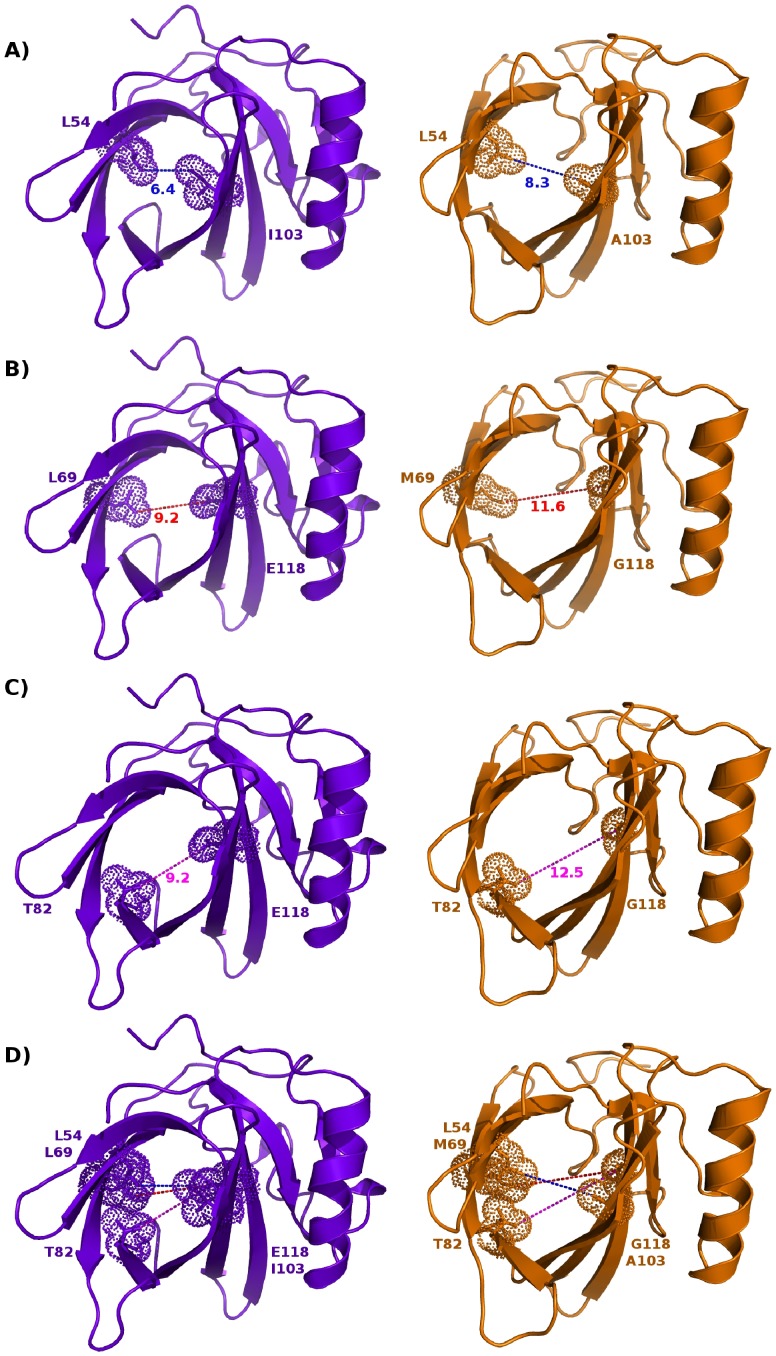
Distance between residues at the centre of the cavity. Cartoon representation of darcin (mauve) and MUP11 (orange) with selected residues at the centre of the beta barrel. Closest distance (in Å) between non-hydrogen atoms is measured between amino acids L54 Cδ1 and I103 Cδ1 (A), L69 Cδ1 and E118 Cδ (B) and T82 Cγ1 and E118 Cδ (C) for darcin and L54 Cδ1 and A103 Cβ (A), M69 Cε and G118 Cα (B) and T82 Cγ1 and G118 Cα (C) for MUP11. The combined effect of these residues on the narrowness/restriction at the centre of the barrel is shown in (D).

The binding selectivity observed here between darcin and MUP11 is likely due to the specific residues lining the cavity. The individual hydrophobic interactions within the cavity either contribute significantly to the free energy of darcin/MUP11 interactions with pheromone or alter the position of the ligand within the cavity to affect the water-mediated hydrogen bonding network. In addition, the features conferred by the individual residues, which are not all conserved, within the cavity of each of the MUPs appear to determine the size and shape of the cavity, and the specific interactions made between protein and pheromones. Fig. 9 shows, for example, how differences at positions 103 and 118 (I103, E118 in darcin for A103/G118 in MUP11) have profound effects on the cavity widths of darcin and MUP11, providing one explanation as to why larger ligands like NPN are not able to bind darcin. Hence, specific residues in the individual MUPs appear to influence pheromone selectivity (and retention), making them the determinants of affinity and specificity, and, therefore, regulating the profiles of pheromones to which each MUP (or class of MUP) can interact with.

### Stability of darcin and MUP11 probed by chemical denaturation

**Figure 10:**
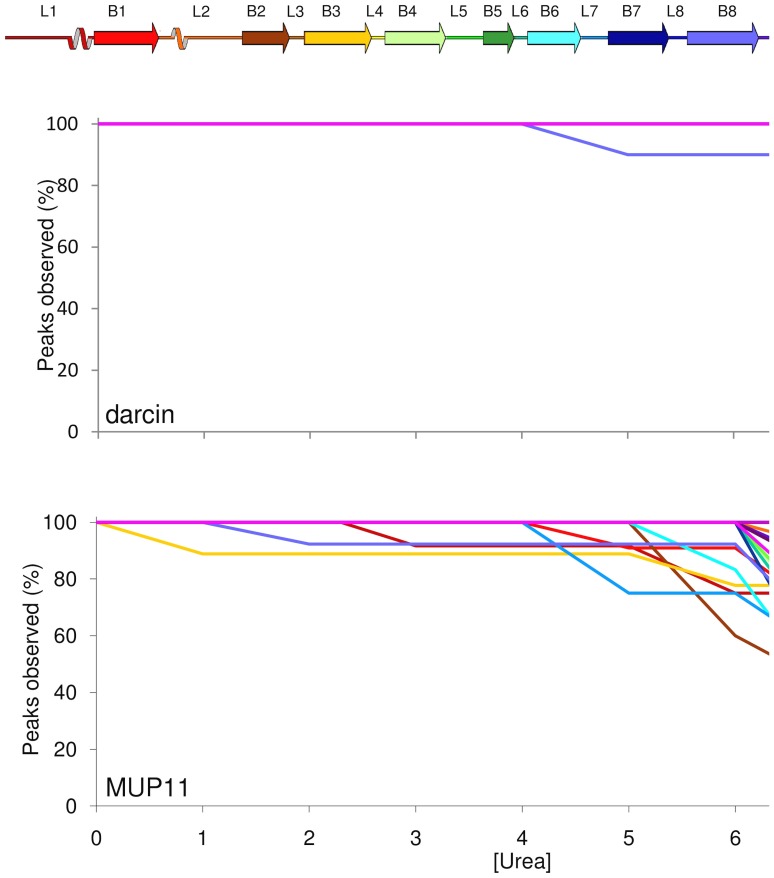
Urea denaturation of darcin and MUP11. (Top) Schematic of secondary structures; loops (L), beta strands (B), and alpha helix (H) of darcin and MUP11. (Middle and Bottom) Plots of % of native backbone NH peaks observed at different urea concentrations for each secondary structure element in darcin (middle) and MUP11 (bottom); secondary structures are coloured coded as in the schematic. In darcin, the only region that is destabilised by urea is L8.

Urinary MUPs, including darcin, have evolved to be deposited in the external environment to play a number of roles in scent signalling. It might be expected that MUPs would exhibit a structural stability commensurate with these roles. To test this, both proteins were titrated with stepwise increases in urea concentration and ^1^H-^15^N HSQC recorded at each interval. As MUP 11 was exposed to higher and higher concentrations of urea, there were minor unfolding events, namely, the shortening of strands β2, β3 and β8 of the beta barrel until 6 M urea, at which point the structure unfolded more extensively, consistent with the loss of beta sheet and helical structures. By contrast, darcin exhibited virtually no structural perturbation even at 7.5 M urea; only one small loop (L_8_) showed evidence of destabilisation above 5 M urea. Over 90% of darcin backbone amide resonances are observable in the ^1^H-^15^N HSQC spectrum at the highest urea concentration, compared with only 45% of the backbone amides resonances in MUP11, confirming the greater preservation of the native darcin structure compared with MUP11 (Figs. 10 and Fig. S2 in File S1). Urea is a denaturant that works by destabilising the hydrogen bond network of a protein. In a beta barrel structure it is anticipated that such a denaturant would have a dramatic impact on the stability, and indeed appears to do so on MUP11. Darcin, on the other hand, appears protected from this destabilising effect. There is 80% sequence identity between both proteins and the most significant structural difference between them is the presence of 3_10_ helix near the N-terminus of darcin which is absent in MUP11 and a better-defined β-strand 5 in darcin compared with MUP11. Detailed analyses show little difference in the intraprotein hydrophobic and ionic interactions between the two proteins. More investigations are hence required to establish the significantly higher stability of darcin.

## Discussion

We present here the structure, physico-chemical and binding characteristics of the peripheral MUP darcin, which is a male-specific pheromone that plays a key role in mouse sexual attraction. We compare darcin with a central MUP, MUP11, which was analysed using the same methodologies. Darcin and MUP11 are both eight-stranded beta barrel proteins. Four short hairpin loops are found at the N-terminal, top end of the barrel whereas larger loops are found at the other C-terminal, bottom end (Fig. 2). The closest distance is between L_1_ and L_5_ at the C-terminal end, as in other MUP structures, with L_1_ occluding the bottom, C terminal end of the internal hydrophobic cavity. The enclosed hydrophobic cavity is similar to other MUPs and, more generically, to lipocalins.

Despite overall structural similarities between darcin and MUP11, there are variations in the non-conserved residues that could explain the differences in the physico-chemical properties between darcin and MUP11. Against SBT, darcin binds with a 20-fold higher affinity than MUP11 and this is, in part, due to the carboxyl side-chain of E118 in darcin being able to form a direct hydrogen bond with the SBT, similar to the structure described for MUP4 [19]; in MUP11, the corresponding residue is G118, leading to a different configuration of the hydrogen bonds that are necessary for SBT binding. The darcin hydrophobic cavity is 11% smaller than MUP11. The non-conserved residues such as E118 (darcin)/G118 (MUP11), I103/A103 appear to also affect the width of the cavity, with the reduced width in darcin being one factor which precludes its binding to larger ligands such as NPN.

Of greatest surprise was the observation of the extreme stability of darcin to denaturation by urea, supported by the lower degree of protonation in electrospray ionisation and the high mobility on SDS-PAGE. All of these properties are consistent with darcin having a stable, compact shape that is very resistant to unfolding, whether in the presence of urea in the solution phase, in the gas phase as a multiply-charged ion, or in the presence of SDS during gel electrophoresis.

The extreme stability of darcin, together with its ability to bind strongly to, and possibly retain, SBT appear to contribute to its biological characteristics. The rate of release of bound pheromones can modulate the time frame of response to scent marks [17]. When MUPs in male mouse urine are resolved by ion exchange chromatography, the binding of SB2HT was predominantly associated with MUP7 and darcin [28], consistent with a degree of specificity in ligand affinity/release. The same studies also showed that MUP-bound SB2HT is displaced much more slowly from dried solid urine than the unbound form. Thus, the extreme stability of darcin may function to retain structural integrity and tight binding of pheromonal ligands in order to delay their release. The urea concentration in freshly voided mouse urine is approximately 0.5 to 0.8 M. At this concentration, neither darcin nor MUP11 would undergo extensive unfolding, and differences in stability would not be critical. However, following deposition of urine, water evaporates rapidly from it. Urea concentration in the residual drying sample will increase rapidly and the data here shows that MUP11 would start to undergo substantial disturbance to its secondary structure ahead of darcin during this drying process; in fact, even at approaching 8 M urea, darcin retains much of its native structure, clearly demonstrating that the stability of darcin is important for prolonging the longevity of volatile SB2HT in urine scent marks.

Darcin is the first protein for which there is clear evidence of pheromonal activity of the protein in its own right. In the urine of a male mouse, darcin stimulates the instinctive attraction of females to spend time where males have deposited their scent [29] whilst also inducing females to learn a preference for spatial location cues where they have encountered darcin [30]. If the intrinsic darcin signal in a male's scent marks degraded faster than other components that signal the individual identity of the scent owner (a signal that involves the central MUPs [21]), this could falsely indicate that the owner of the scent had low or non-existent production of this key pheromone, making the male unattractive to females. There is, therefore, likely to be particularly strong selection on darcin to ensure that it is highly stable and of greater persistence than any components signalling the identity of the scent owner. Darcin is also effective in stimulating female attraction even when encountered without other volatile or involatile components of male urine; hence its high stability makes sense since the prolonged persistence of darcin is necessary for it to continue to attract females to a scent marked site.

The MUP gene cluster has been something of an enigma. It has not been entirely clear why so many MUPs are required to create a simple, pheromone binding/release system. Moreover, the identification of more highly conserved central MUPs and more distinct peripheral MUPs has suggested multiple functions. The structural characterisation of darcin has revealed remarkable properties that set it apart from central MUPs and which can be readily rationalised in the context of known functions. Whether such uniqueness extends to other MUPs is a question that remains to be addressed.

This study reports the first experimental evidence showing the unusual stability of darcin. The high stability of darcin and its high affinity towards SBT support the notion that darcin binds tightly to and retains certain pheromones, and, thereby, is able to sequester and transport these small, volatile molecules to the receptor.

## Supporting Information

File S1 **Figure S1, MUP11 and darcin relaxation dynamics.** A comparison of MUP11 and Darcin relaxation data and RCI to determine differing flexibilities. (Top) Darcin and MUP11 relaxation at 600 MHz, T_1_/T_2_ in blue (Darcin) and orange (MUP11), (Middle) heteronuclear NOEs in blue (Darcin) and orange (MUP11). (Bottom) RCI analysis; Darcin (blue) and MUP11 (orange) random coil index. Secondary structure cartoon representation of darcin is shown at the top of each panel. **Figure S2, Chemical Shift Perturbations induced upon urea denaturation.** ^1^H ^15^N HSQC spectra of darcin (left) and MUP11 (right) in the absence (red) and presence (blue) of 7.5 M urea. Urea induces limited shift perturbations in the darcin spectrum whereas large chemical shift changes can be observed in the MUP11 spectrum. **Table S1, Beta-barrel cavity residues.** Strand position and location of the cavity core residues in the beta-barrel.
(DOC)
