# Supplementary material for: The Structure, Stability and Pheromone Binding of the Male Mouse Protein Sex Pheromone Darcin
Source: PLoS One. 2014 Oct 3;9(10):e108415. doi: 10.1371/journal.pone.0108415 (PMC4184797; doi:10.1371/journal.pone.0108415)
Supplement: File S1 — Figure S1, MUP11 and darcin relaxation dynamics. A comparison of MUP11 and Darcin relaxation data and RCI to determine differing flexibilities. (Top) Darcin and MUP11 relaxation at 600 MHz, T1/T2 in blue (Darcin) and orange (MUP11), (Middle) heteronuclear NOEs in blue (Darcin) and orange (MUP11). (Bottom) RCI analysis; Darcin (blue) and MUP11 (orange) random coil index. Secondary structure cartoon representation of darcin is shown at the top of each panel. Figure S2, Chemical Shift Perturbations induced upon urea denaturation. 1H 15N HSQC spectra of darcin (left) and MUP11 (right) in the absence (red) and presence (blue) of 7.5 M urea. Urea induces limited shift perturbations in the darcin spectrum whereas large chemical shift changes can be observed in the MUP11 spectrum. Table S1, Beta-barrel cavity residues. Strand position and location of the cavity core residues in the beta-barrel. (DOC) [file pone.0108415.s001.doc]

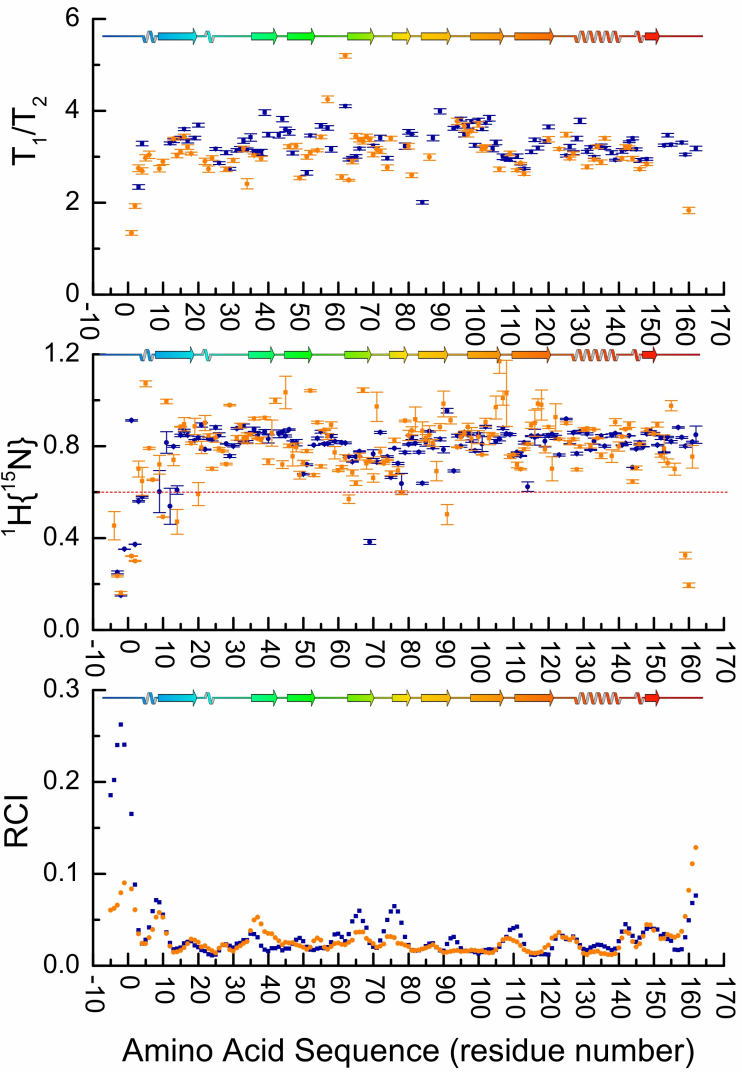


**Figure S1. MUP11 and darcin relaxation dynamics.** A comparison of MUP11 and Darcin relaxation data and RCI to determine differing flexibilities. (Top) Darcin and MUP11 relaxation at 600MHz, T_1_/T_2_ in blue (Darcin) and orange (MUP11), (Middle) heteronuclear NOEs in blue (Darcin) and orange (MUP11). (Bottom) RCI analysis; Darcin (blue) and MUP11 (orange) random coil index. Secondary structure cartoon representation of darcin is shown at the top of each panel.

**Table S1. Beta-barrel cavity residues.** Strand position and location of the cavity core residues in the beta-barrel.

|  | **Central** | | **N terminal, top end** | | **C terminal, bottom end** | |
| --- | --- | --- | --- | --- | --- | --- |
| strand | MUP11 | darcin | MUP11 | darcin | MUP11 | darcin |
| β1 | NONE | NONE | NONE | NONE | L24 | L24 |
| β2 | L42 | V42 | I45 | I45 | F38 & L40 | M38 & V40 |
| β3 | L54 | L54 | L52 | L52 | F56 | F56 |
| β4 | M69 | L69 | NONE | NONE | NONE | NONE |
| β5 | V82 | L82 | NONE | NONE | Y84 | Y84 |
| β6 | F90 | F90 | I92 | I92 | N88 | N88 |
| β7 | A103 | I103 | L101 | I101 | L107 | L107 |
| β8 | G118 | E118 | Y120 | Y120 | L116 | L116 |

**
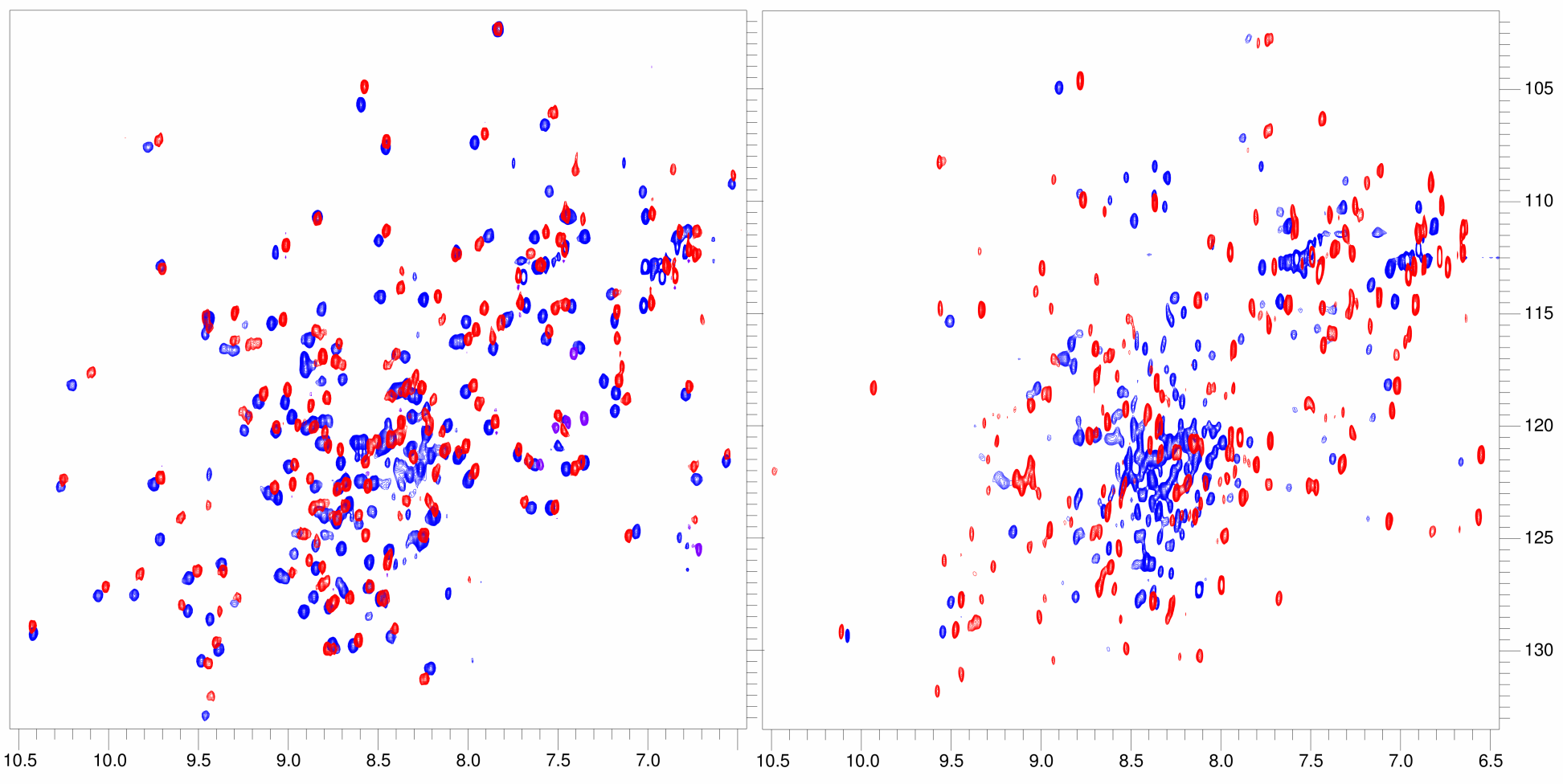
**

**Figure S2.** **Chemical Shift Perturbations induced upon urea denaturation.** ^1^H ^15^N HSQC spectra of darcin (left) and MUP11 (right) in the absence (red) and presence (blue) of 7.5M urea. Urea induces limited shift perturbations in the darcin spectrum whereas large chemical shift changes can be observed in the MUP11 spectrum.
